# The prevalence and determinant of overweight and obesity among residents aged 40–69 years in high-risk regions for upper gastrointestinal cancer in southeast China

**DOI:** 10.1038/s41598-023-35477-x

**Published:** 2023-05-20

**Authors:** Xiang Feng, Jinhua Zhu, Zhaolai Hua, Qiuping Shi, Jinyi Zhou, Pengfei Luo

**Affiliations:** 1 Institute of Tumour Prevention and Control, Yangzhong People’s Hospital, Yangzhong, 212200 China; 2 Department of Non-communicable Disease Prevention and Control, Jiangsu Provincial Centre for Disease Control and Prevention, Nanjing, 210009 China; 3 Department of Gastroenterology, Zhongda Hospital, Southeast University, Nanjing, 210000 China

**Keywords:** Health care, Risk factors

## Abstract

Being overweight or obese is one of the public health concerns worldwide, and its prevalence is gradually increasing. Obesity has been proven to be associated with some cancers, including upper gastrointestinal cancer (UGC). However, studies on the prevalence of obesity among residents of areas at high risk of UGC in China are minimal. The aim of this study is to assess the prevalence of obesity and its influencing factors among people aged 40–69 years (high-risk population) in high-risk areas for UGC in Jiangsu Province, southeast China. This cross-sectional study involved 45,036 subjects aged 40–69 years identified in the Rural Early Diagnosis and Treatment of UGC Project database in Jiangsu Province from 2017 to 2021. Differences in prevalence across gender and age were assessed using the Chi-square test. Using a multinomial logistic regression model, we examined independent risk factors for overweight/obesity and their gender and age differences. The prevalence of overweight, obesity, and overweight/obesity varied based on the standards used: Chinese standard (42.1%, 11.9%, and 54.0%) and WHO standard (34.7%, 4.7%, and 39.4%), respectively. Being overweight was more common in men than women, while obesity was more common in women than men. Age of 50–59 years, married, household size of 7–9, drinking, soy products, pickled food, and hot food intake were positively associated with overweight/obesity. Females, 60–69 years, higher education level, household size of 4–6, annual family income of more than 60,000 CNY, smoking, and fresh fruit intake were negatively associated with overweight/obesity. Stratified analysis showed that the effects of age, education and meat, egg and dairy products on overweight/obesity were different across gender. The impact of fresh fruit and vegetables on overweight/obesity was also heterogeneous between the younger (40–59 years) and older (60–69 years) groups. In conclusion, the prevalence of overweight and obesity is high among adults aged 40–69 years from high-risk areas for UGC of Jiangsu Province, southeast China. Independent influencing factors of being overweight/obese included gender, age, marital status, education, household size, annual family income, smoking, drinking, fresh fruit, soy products, pickled food and hot food intake, and may vary by gender and age. Screening-based interventions should be considered to control obesity levels among screened participants. Besides, heterogeneity of influencing factors across subgroups could be focused on to improve intervention effectiveness.

## Introduction

Obesity has become a critical public health problem for the health of populations worldwide. It has been treated as a disease^1,2^. In 2015, according to World Health Organization (WHO), 107.7 million children and 603.7 million adults were reported to be obese globally, with a prevalence of 5.0% and 12.0%, respectively^3^. The prevalence of obesity has doubled in 73 countries since 1980 and continues to increase in most countries^3,4^. Correspondingly, the burden of obesity is enormous. In 2015, high body mass index (BMI) was associated with 4 million deaths worldwide, losing 120 million disability-adjusted life years^3^. When overweight is included in the calculation, the burden of disease associated with excess weight in 2014 was estimated to be approximately US$2.0 trillion^5^. As a country with a large population, China has the highest number of obese adults and children globally. It is estimated that 85 million people were obese in 2018, with the standardized obesity rate for adults increasing nearly twofold between 2004 and 2018^6^. Moreover, in 2016, approximately one-third of Chinese adolescents were overweight or obese^7^.

As the global obesity epidemic has reached alarming levels, scholars have studied various aspects of overweight and obesity and found that they are associated with gastric cancer (GC) and oesophageal cancer (OC)^8,9^. For instance, Song et al.^10^ found an association between obesity at age 18 and GC. They revealed that the risk of GC increased when BMI was 25.3 kg/m^2^ and above, in both males and females, compared with their counterparts. Wang et al.^11^ studied the US population’s attributable risk of GC and OC. They found that obesity and gastroesophageal reflux disease were associated with more than 1/2 of oesophageal adenocarcinomas and 1/3 of gastric cardia adenocarcinomas. Although the epidemiological evidence for obesity and GC and OC may vary somewhat depending on the type of cancer, there is ample evidence that obesity is a risk factor for upper gastrointestinal cancer (UGC)^12,13^. Due to the implementation of cancer prevention and control measures in various countries, GC and OC incidences have declined in recent years. However, they are still prevalent in Asia, especially in China. GC and OC can be found in the Chinese top six cancer incidence and mortality rates^14,15^.

Except for the UGC, obesity is also thought to contribute to some common chronic diseases and can cause co-morbidities^4,7,16^. Hence, people who are obese will be at greater health risk. In particular, when individuals simultaneously have multiple health risk factors, interactive effects between factors will significantly increase the risk of disease^17,18^. In China, many scholars have studied the prevalence of different obesity indicators in adults and children. They found a general prevalence of obesity/overweight and that the prevalence and influencing factors vary between subgroups^4,7,16,19,20^. However, few studies have been conducted on specific populations. For example, no studies have examined the prevalence of obesity among people at high risk of UGC. This has led to a lack of epidemiological basis for obesity management of UGC and other common chronic diseases in this group. Therefore, we used screening data from the Rural Early Diagnosis and Treatment of UGC in Jiangsu Province to assess the prevalence of overweight and obesity among individuals aged 40–69 years and analysis its associated factors. In addition, we explored the sex and age differences of associated factors, aiming to provide a reference for health policies and public health interventions that would promote weight management among the target population in Jiangsu Province.

## Methods

### Study design and population

**Figure 1:**
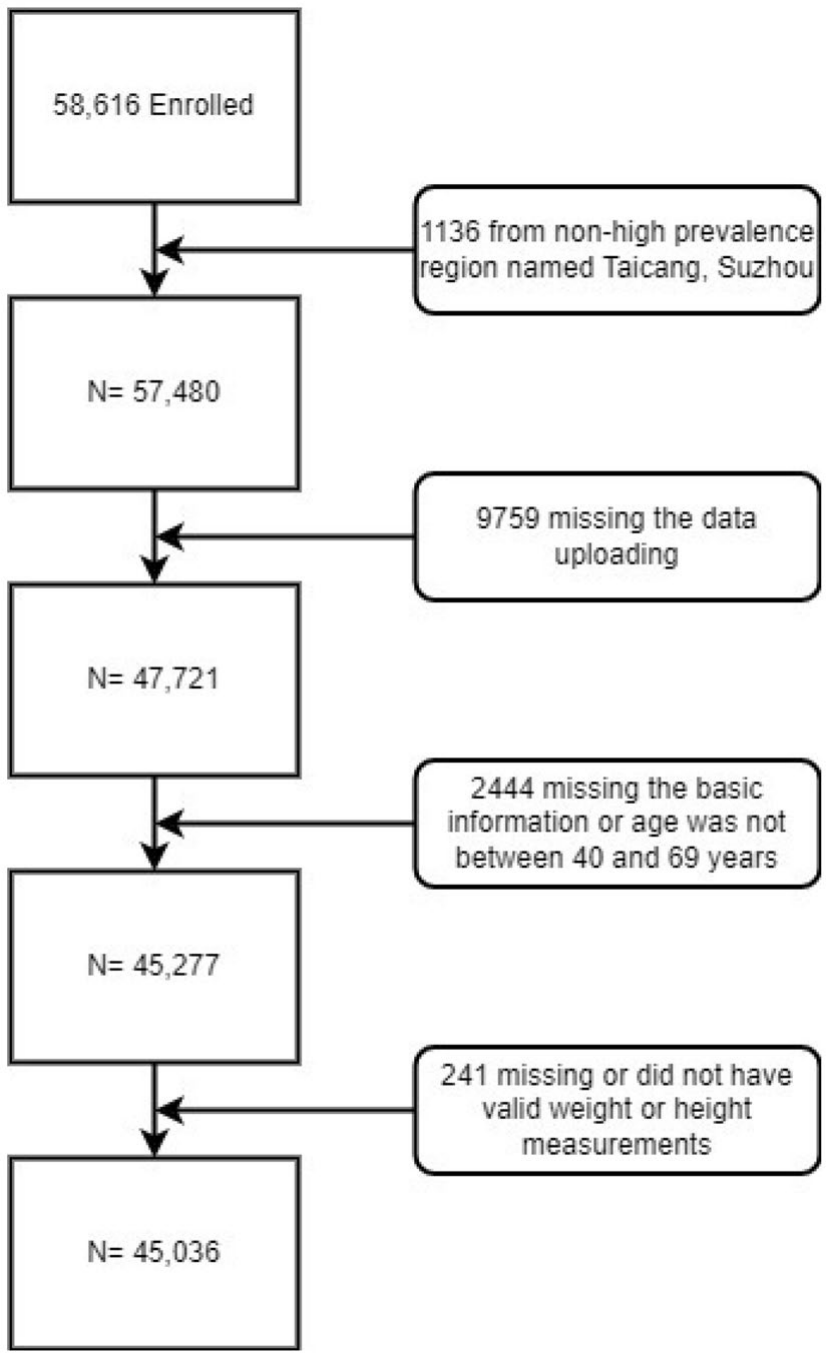
Flow diagram of the study participants included.

The data for this cross-sectional study were obtained from the Rural Early Diagnosis and Treatment of UGC Project in Jiangsu Province from January 2017 to August 2021. The project followed the technical protocol for screening and early diagnosis and treatment of UGC (Trial Version 2020), from population selection, inclusion/exclusion criteria, screening process, and diagnostic criteria to follow-up method^21^. Briefly, the project unit from Jiangsu Province, in an area with a high incidence of UGC, identifies townships or villages with a high incidence of GC and OC as the screening regions by referring to local cancer incidence data. The cluster sampling method was adopted to invite residents aged 40–69 years (treated as a high-risk population for UGC in China) in the regions to participate in UGC screening unless they had a non-compliant age, were unwilling to participate, had contraindications to endoscopy or had a history of the UGC/mental disorder^21,22^. The aim is to perform endoscopy and pathological diagnosis among participants to detect early disease of the UGC as far as possible and thus provide intervention to reduce the incidence and mortality of GC and OC^22^. Each project site must screen 1000–2000 high-risk individuals per year. 58,616 participants from more than 84 townships/subdistricts in twelve counties/districts across six cities (Huaian, Suzhou, Taizhou, Yancheng, Yangzhou, and Zhenjiang) in Jiangsu Province were included. Of these participants, we excluded 1136 from non-high prevalence areas, 9759 whose screening data were not uploaded, 2444 whose basic information was missing or whose age did not match, and 241 whose standard height or weight measurements were missing. Ultimately, 45,036 were included in the analysis (Fig. 1). The research followed the Declaration of Helsinki. It was approved by the academic and ethical committee of the Yangzhong People's Hospital (Approval number 202152). All participants involved in the study signed an informed consent form.

### Study procedure and data collection

The main screening process for UGC includes informed consent, screening registry, physical measurements, basic information survey, endoscopy and biopsy pathology. The data used in this study were extracted from the information questionnaire. The questionnaire includes the following items: (1) general information (gender, age, marital status, education, household size, annual family income), (2) physical measurements (height, weight, pulse and blood pressure), (3) lifestyle and dietary characteristics (smoking, drinking, frequency of consumption of vegetables, fruit, meat, egg and milk, soy products, pickled, fried, hot and mouldy food), (4) history of digestive system disease. Through one-to-one, face-to-face visits, well-trained physicians, nurses, and epidemiological investigators administered the forms after participants completed the informed consent, screening registry and physical measurements. The staff did not give any hints about the answers to the questions during this period. The entire survey took 10–15 min. Height and weight measurements conducted by trained researchers followed standard methods with the subjects in light indoor clothing without shoes and hats^4,7,16,19,22^. Height was accurately measured to 0.1 cm, and body weight was accurate to 0.1 kg. The earliest project sites included in this study were enrolled in 2006 and the latest in 2012, so all the staff have extensive experience in epidemiological investigations. Besides, all investigators must attend regular training and assessment organized by the Expert Group on Rural Early Diagnosis and Treatment of UGC to remain well-trained. The questionnaire included was also designed by experts, and the items are simple and easy to understand (Supplementary Table 1). After the survey, quality was controlled by dedicated staff. Missing or logical errors were added or corrected on the spot. After ensuring the data's authenticity and validity, dedicated staff entered it into the online system. The uploaded data was also regularly reviewed and summarised by provincial experts.

### Definitions

Obesity is measured by calculating BMI as weight/height squared (kg/m^2^). Although it is less valuable than waist circumference or waist-to-hip ratio in describing abdominal obesity, it is still widely used^23^. The criteria for classifying BMI vary between organizations, with the WHO using 18.5, 25 and 30 as the cut-off points (underweight: < 18.5 kg/m^2^; normal: 18.5–24.9 kg/m^2^; overweight: 25.0–29.9 kg/m^2^; obesity: BMI ≥ 30.0 kg/m^2^)^24^. In the Chinese population, 18.5, 24 and 28 are recommended as cut-off points (underweight: < 18.5 kg/m^2^; normal: 18.5–23.9 kg/m^2^; overweight: 24–27.9 kg/m^2^; obesity: BMI ≥ 28.0 kg/m^2^)^25^. Therefore, this study presents the situation according to two criteria when describing the prevalence of overweight and obesity.

Sociodemographic variables included gender (male, female), age (40–49 years, 50–59 years, 60–69 years), marital status (married, never married/divorced/separated/widowed), education (illiterate or semi-illiterate, primary school, secondary school, high school and above), household size (number of persons in the family: 0–3, 4–6, 7–9), annual family income [< 29,999 Chinese Yuan (CNY), 30,000–59,999 CNY and ≥ 60,000 CNY]. Smoking was classified as “Yes ” (participants self-reported having consistent smoking habits, regardless of the type of tobacco product) and “No”. Drinking was classified as “Yes ” (participants self-reported having drinking habits consistently, regardless of the type of alcohol) and “No”. Intake of fresh vegetables, fresh fruit, meat, egg and milk, soy products, and pickled, fried, hot, mouldy food classified as “Yes ” were defined as subjects who had eaten them more than once a week in the past year.

### Statistical analysis

Mean and standard deviation (SD), frequency and percentage describe the characteristics of the participants and the prevalence of overweight and obesity, where appropriate, respectively. The *χ*^*2*^ test was used to compare the prevalence of overweight and obesity across gender and age, with *P* < 0.05 considered to be significant. We used a multinomial logistic regression model to explore the independent influences of overweight and obesity, with risks presented as crude odds ratio (COR) /adjusted odds ratio (AOR) and 95% confidence interval (CI). The dependent variable was classified as underweight/normal (reference), overweight and obesity. We implemented not only a total population analysis but also a sex-and age-stratified analysis, as we considered that the predictors or associations might differ by gender and age. The final regression models were adjusted for all variables, and only variables with *P* < 0.05 were considered statistically significant. All statistical analyses were performed by SPSS version 26.0.

## Results

### Basic characteristics of the participants

A total of 45,036 screening subjects aged 40–69 years (mean age: 56.7 ± 7.6 years) were included in the analysis, with an effective rate of 76.8% (Table 1). Of the 45,036 individuals, 55.9% were female, 40.8% were aged 60–69 years, 94.6% were married, 36.1% had the education level of secondary school, and most (54.7%) had 4–6 family members. 30,000–59,999 CNY was the most common annual family income. Furthermore, a minority of participants smoked (19.3%) and consumed alcohol (17.2%). Among the different dietary habits, the proportion of regular intake of fresh vegetables (96.6%), fruit (63.5%), meat, egg and milk (72.5%), and soy products (69.7%) was higher. The majority of the participants had no habit of consuming pickled (84.6%), fried (97.0%), hot (94.3%) and mouldy (99.7%) food.

**Table 1:** Basic information, lifestyle and dietary behaviour of participants.

Variable	Category	N	%
Gender	Male	19,855	44.1
	Female	25,181	55.9
Age (years, mean ± SD)	56.7 ± 7.6		
Age group (years)	40–49	8846	19.6
	50–59	17,799	39.5
	60–69	18,391	40.8
Marital status	Married	42,583	94.6
	Other^a^	2453	5.4
Education	Illiterate or semi-illiterate	8287	18.4
	Primary school	14,922	33.1
	Secondary school	16,273	36.1
	High school and above	5554	12.3
Household size	0–3	18,180	40.4
	4–6	24,635	54.7
	7–9	2221	4.9
Annual family income (CNY)	< 29,999	13,953	31.0
	30,000–59,999	15,688	34.8
	≥ 60,000	15,395	34.2
Smoking	No	36,360	80.7
	Yes	8676	19.3
Drinking	No	37,268	82.8
	Yes	7768	17.2
Fresh vegetables	No	1517	3.4
	Yes	43,519	96.6
Fresh fruit	No	16,450	36.5
	Yes	28,586	63.5
Meat, egg and milk	No	12,374	27.5
	Yes	32,662	72.5
Soy products	No	13,626	30.3
	Yes	31,410	69.7
Pickled food	No	38,115	84.6
	Yes	6921	15.4
Fried food	No	43,685	97.0
	Yes	1351	3.0
Hot food	No	42,459	94.3
	Yes	2577	5.7
Mouldy food	No	44,912	99.7
	Yes	124	0.3

*SD* standard deviation, *CNY* Chinese Yuan.

^a^Never married/divorced/separated/widowed.

### Prevalence of overweight and obesity

The prevalence of overweight and obesity classified by BMI in different age and gender groups based on different standards is presented in Table 2. Using the Chinese reference, the prevalence of overweight and obesity was 42.1% (44.1% for men and 40.5% for women) and 11.9% (11.5% for men and 12.3% for women), respectively. Based on the WHO reference, the prevalence of overweight and obesity was 34.7% (36.3% for men and 33.5% for women) and 4.7% (4.1% for men and 5.2% for women), respectively. Regardless of the standard, the proportion of overweight was higher in men than in women, but the reverse was true for the obese population (*P* < 0.001). There were also significant age differences in the prevalence of overweight and obesity (*P* < 0.001). The highest proportion of participants categorized as overweight or obese was observed in the 50–59 age group when all two classifications were used.

**Table 2:** The prevalence of overweight and obesity among participants classified by BMI of different ages and genders.

Age (years)	N	BMI (kg/m^2^)^a^				BMI (kg/m^2^)^b^			
		Underweight	Normal weight	Overweight	Obesity	Underweight	Normal weight	Overweight	Obesity
Men									
40–49	3511	21 (0.6)	1368 (39.0)	1642 (46.8)	480 (13.7)	21 (0.6)	1907 (54.3)	1415 (40.3)	168 (4.8)
50–59	7152	65 (0.9)	2911 (40.7)	3303 (46.2)	873 (12.2)	65 (0.9)	4020 (56.2)	2759 (38.6)	308 (4.3)
60–69	9192	166 (1.8)	4298 (46.8)	3802 (41.4)	926 (10.1)	166 (1.8)	5645 (61.4)	3035 (33.0)	346 (3.8)
Total	19,855	252 (1.3)	8577 (43.2)	8747 (44.1)	2279 (11.5)	252 (1.3)	11,572 (58.3)	7209 (36.3)	822 (4.1)
Women									
40–49	5335	91 (1.7)	2680 (50.2)	1974 (37.0)	590 (11.1)	91 (1.7)	3379 (63.3)	1618 (30.3)	247 (4.6)
50–59	10,647	124 (1.2)	4729 (44.4)	4446 (41.8)	1348 (12.7)	124 (1.2)	6285 (59.0)	3665 (34.4)	573 (5.4)
60–69	9199	181 (2.0)	4084 (44.4)	3779 (41.1)	1155 (12.6)	181 (2.0)	5397 (58.7)	3144 (34.2)	477 (5.2)
Total	25,181	396 (1.6)	11,493 (45.6)	10,199 (40.5)	3093 (12.3)	396 (1.6)	15,061 (59.8)	8427 (33.5)	1297 (5.2)
All subjects									
40–49	8846	112 (1.3)	4048 (45.8)	3616 (40.9)	1070 (12.1)	112 (1.3)	5286 (59.8)	3033 (34.3)	415 (4.7)
50–59	17,799	189 (1.1)	7640 (42.9)	7749 (43.5)	2221 (12.5)	189 (1.1)	10,305 (57.9)	6424 (36.1)	881 (4.9)
60–69	18,391	347 (1.9)	8382 (45.6)	7581 (41.2)	2081 (11.3)	347 (1.9)	11,042 (60.0)	6179 (33.6)	823 (4.5)
Total	45,036	648 (1.4)	20,070 (44.6)	18,946 (42.1)	5372 (11.9)	648 (1.4)	26,633 (59.1)	15,636 (34.7)	2119 (4.7)

Data are presented as n (%). Chi-square test was performed to test the differences between genders and ages.

*BMI* body mass index.

^a^Adopting the Chinese standard (overweight: 24 kg/m^2^ ≤ BMI < 28 kg/m^2^; obesity: BMI ≥ 28 kg/m^2^).

^b^Adopting the WHO standard (overweight: 25 kg/m^2^ ≤ BMI < 30 kg/m^2^; obesity: BMI ≥ 30 kg/m^2^).

### Factors for overweight/obesity

Table 3 shows the univariate and multivariate logistic regression analysis of factors associated with overweight or obesity. In this study, all potential covariates were included in the final multivariate analysis. After complete adjustments, the results show that females were less likely to be overweight (AOR = 0.79, 95% CI 0.75, 0.83) and obese (AOR = 0.80, 95% CI 0.74, 0.87) than males. Compared with participants aged 40 to 49 years, individuals aged 50 to 59 years were at greater risk of being overweight (AOR = 1.12, 95% CI 1.06, 1.19), while those aged 60 to 69 years were at lower risk of being obese (AOR = 0.74, 95% CI 0.68, 0.81). The odds of being overweight were higher among married participants (AOR = 1.13, 95% CI 1.04, 1.24). Increased levels of education were associated with a decreased risk of being overweight (primary school: AOR = 0.84, 95% CI 0.79, 0.89; secondary school: AOR = 0.86, 95% CI 0.81, 0.92; high school and above: AOR = 0.82, 95% CI 0.75, 0.89) and obese (primary school: AOR = 0.67, 95% CI 0.62, 0.73; secondary school: AOR = 0.58, 95% CI 0.53, 0.64; high school and above: AOR = 0.56, 95% CI 0.49, 0.63). The odds of being obese among respondents having 4 to 6 family members (AOR = 0.94, 95% CI 0.88, 1.00) was lower than those with 0 to 3 family members. However, the odds of obesity increased when individuals had 7 to 9 family members (AOR = 1.28, 95% CI 1.11, 1.46). The odds of being overweight (AOR = 0.91, 95% CI 0.86, 0.96) and obese (AOR = 0.75, 95% CI 0.69, 0.81) among those had higher annual family income (≥ 60,000 CNY) was less than those with lower ones (< 29,999 CNY). Besides, smokers were less likely to be overweight (AOR = 0.71, 95% CI 0.67, 0.75) and obesity (AOR = 0.64, 95% CI 0.59, 0.71) while drinking increased its risk (overweight: AOR = 1.27, 95% CI 1.20, 1.35; obesity: AOR = 1.32, 95% CI 1.20, 1.45). The protective effect of fresh fruit was only significant for obesity (AOR = 0.91, 95% CI 0.84, 0.98). The AOR of overweight and obesity was increased for those who had soy products (overweight: AOR = 1.06, 95% CI 1.01, 1.12; obesity: AOR = 1.13, 95% CI 1.04, 1.23), pickled (overweight: AOR = 1.20, 95% CI 1.13, 1.27; obesity: AOR = 1.53, 95% CI 1.41, 1.67) and hot food (overweight: AOR = 1.11, 95% CI 1.01, 1.21; obesity: AOR = 1.18, 95% CI 1.04, 1.34) in comparison to those without these habits.

**Table 3:** Factors associated with the prevalence of overweight or obesity among participants.

Variable	Overweight				Obesity			
	COR (95%CI)	*P*-value	AOR (95%CI)	*P*-value	COR (95%CI)	*P*-value	AOR (95%CI)	*P*-value
Gender								
Male	1.00		1.00		1.00		1.00	
Female	0.87 (0.83, 0.90)	< 0.001	0.79 (0.75, 0.83)	< 0.001	1.01 (0.95, 1.07)	0.800	0.80 (0.74, 0.87)	< 0.001
Age group (years)								
40–49	1.00		1.00				1.00	
50–59	1.14 (1.08, 1.20)	< 0.001	1.12 (1.06, 1.19)	< 0.001	1.10 (1.02, 1.20)	0.019	1.02 (0.94, 1.11)	0.658
60–69	1.00 (0.95, 1.05)	0.975	0.95 (0.90, 1.01)	0.099	0.93 (0.85, 1.01)	0.071	0.74 (0.68, 0.81)	< 0.001
Marital status								
Other^a^	1.00		1.00				1.00	
Married	1.14 (1.04, 1.24)	0.004	1.13 (1.04, 1.24)	0.006	1.05 (0.92, 1.20)	0.452	1.12 (0.98, 1.28)	0.102
Education								
Illiterate or semi-illiterate	1.00		1.00				1.00	
Primary school	0.89 (0.84, 0.94)	< 0.001	0.84 (0.79, 0.89)	< 0.001	0.69 (0.64, 0.75)	< 0.001	0.67 (0.62, 0.73)	< 0.001
Secondary school	0.97 (0.91, 1.02)	0.230	0.86 (0.81, 0.92)	< 0.001	0.65 (0.60, 0.70)	< 0.001	0.58 (0.53, 0.64)	< 0.001
High school and above	0.94 (0.87, 1.01)	0.076	0.82 (0.75, 0.89)	< 0.001	0.62 (0.56, 0.69)	< 0.001	0.56 (0.49, 0.63)	< 0.001
Household size								
0–3	1.00		1.00				1.00	
4–6	0.99 (0.95, 1.03)	0.653	1.01 (0.96, 1.05)	0.753	0.90 (0.85, 0.96)	0.001	0.94 (0.88, 1.00)	0.046
7–9	1.11 (1.01, 1.22)	0.029	1.09 (0.99, 1.21)	0.069	1.32 (1.15, 1.50)	< 0.001	1.28 (1.11, 1.46)	0.001
Annual family income (CNY)								
< 29,999	1.00		1.00				1.00	
30,000–59,999	1.03 (0.98, 1.08)	0.306	1.00 (0.95, 1.05)	0.883	0.96 (0.90, 1.04)	0.312	0.95 (0.89, 1.03)	0.215
≥ 60,000	0.93 (0.88, 0.98)	0.003	0.91 (0.86, 0.96)	< 0.001	0.74 (0.68, 0.79)	< 0.001	0.75 (0.69, 0.81)	< 0.001
Smoking								
No	1.00		1.00				1.00	
Yes	0.88 (0.84, 0.93)	< 0.001	0.71 (0.67, 0.75)	< 0.001	0.78 (0.72, 0.84)	< 0.001	0.64 (0.59, 0.71)	< 0.001
Drinking								
No	1.00		1.00				1.00	
Yes	1.22 (1.16, 1.28)	< 0.001	1.27 (1.20, 1.35)	< 0.001	1.15 (1.06, 1.24)	0.001	1.32 (1.20, 1.45)	< 0.001
Fresh vegetables								
No	1.00		1.00				1.00	
Yes	1.01 (0.91, 1.13)	0.850	0.94 (0.84, 1.05)	0.256	1.08 (0.91, 1.28)	0.369	1.05 (0.88, 1.25)	0.602
Fresh fruit								
No	1.00		1.00				1.00	
Yes	1.06 (1.02, 1.11)	0.005	1.03 (0.98, 1.08)	0.327	0.97 (0.91, 1.03)	0.305	0.91 (0.84, 0.98)	0.015
Meat, egg and milk								
No	1.00		1.00				1.00	
Yes	1.12 (1.07, 1.17)	< 0.001	1.04 (0.98, 1.11)	0.157	1.15 (1.08, 1.23)	< 0.001	1.07 (0.98, 1.17)	0.112
Soy products								
No	1.00		1.00				1.00	
Yes	1.12 (1.08, 1.17)	< 0.001	1.06 (1.01, 1.12)	0.030	1.19 (1.11, 1.27)	< 0.001	1.13 (1.04, 1.23)	0.003
Pickled food								
No	1.00		1.00				1.00	
Yes	1.26 (1.19, 1.33)	< 0.001	1.20 (1.13, 1.27)	< 0.001	1.72 (1.59, 1.86)	< 0.001	1.53 (1.41, 1.67)	< 0.001
Fried food								
No	1.00		1.00				1.00	
Yes	1.15 (1.03, 1.30)	0.016	0.98 (0.87, 1.11)	0.767	1.32 (1.12, 1.56)	0.001	0.95 (0.80, 1.14)	0.594
Hot food								
No	1.00		1.00				1.00	
Yes	1.22 (1.12, 1.33)	< 0.001	1.11 (1.01, 1.21)	0.026	1.51 (1.34, 1.70)	< 0.001	1.18 (1.04, 1.34)	0.009
Mouldy food								
No	1.00		1.00				1.00	
Yes	0.91 (0.62, 1.33)	0.618	0.79 (0.54, 1.17)	0.242	1.05 (0.60, 1.82)	0.873	0.80 (0.46, 1.41)	0.447

Regressions were adjusted for gender, age, marital status, education, household size, annual family income, smoking, drinking, intake of fresh vegetables, fresh fruit, meat, egg and milk, soy products, pickled food, fried food, hot food, and mouldy food. Adopting the Chinese standard (overweight: 24 kg/m^2^ ≤ BMI < 28 kg/m^2^; obesity: BMI ≥ 28 kg/m^2^).

*COR* crude odds ratio, *AOR* adjusted odds ratio, *CI* confidence interval, *CNY* Chinese Yuan.

^a^Never married/divorced/separated/widowed.

### Factors for overweight/obesity among different gender groups

Variables associated with overweight or obesity in the final models included age, education, annual family income, smoking, drinking, and intake of meat, egg, milk, soy products, and pickled food among both males and females (Table 4). Meanwhile, the odds of being overweight were higher among married men (AOR = 1.20, 95% CI 1.05, 1.38). Women with 4 to 6 family members (AOR = 0.88, 95% CI 0.80, 0.96) were less likely to be obese than those with 0 to 3 family members. However, the odds of obesity increased when women had 7 to 9 family members (AOR = 1.32, 95% CI 1.11, 1.58). Men consuming hot food were more likely to be obese (AOR = 1.22, 95% CI 1.00, 1.49) than their counterparts. Besides, we found some patterns worth mentioning from the results mentioned above. First, women (unlike men) aged 50 to 59 years were more likely to be overweight or obese. Women aged 60 to 69 were also more likely to be overweight. Second, education in men was positively associated with being overweight, while its effect was inversely in women. Third, men's meat, egg and milk product intake showed a negative correlation with being overweight. In women, however, it is positively associated with overweight and obesity (all *P* < 0.05).

**Table 4:** Factors associated with the prevalence of overweight or obesity among males and females.

Variable	Male				Female			
	Overweight		Obesity		Overweight		Obesity	
	COR (95%CI)	AOR (95%CI)	COR (95%CI)	AOR (95%CI)	COR (95%CI)	AOR (95%CI)	COR (95%CI)	AOR (95%CI)
Age group (years)								
40–49	1.00	1.00	1.00	1.00	1.00	1.00		1.00
50–59	0.94 (0.86, 1.02)	0.94 (0.86, 1.03)	0.85 (0.75, 0.97)^a^	0.83 (0.73, 0.95)^b^	1.29 (1.20, 1.38)^c^	1.23 (1.15, 1.32)^c^	1.30 (1.17, 1.45)^c^	1.12 (1.00, 1.25)^a^
60–69	0.72 (0.66, 0.78)^c^	0.77 (0.70, 0.84)^c^	0.60 (0.53, 0.68)^c^	0.60 (0.52, 0.68)^c^	1.24 (1.16, 1.34)^c^	1.12 (1.03, 1.21)^b^	1.27 (1.14, 1.42)^c^	0.85 (0.75, 0.96)^a^
Marital status								
Other^d^	1.00	1.00	1.00	1.00	1.00	1.00		1.00
Married	1.33 (1.17, 1.52)^c^	1.20 (1.05, 1.38)^b^	1.25 (1.02, 1.54)^a^	1.15 (0.92, 1.42)	1.00 (0.89, 1.13)	1.06 (0.95, 1.20)	0.93 (0.78, 1.10)	1.05 (0.89, 1.25)
Education								
Illiterate or semi-illiterate	1.00	1.00	1.00	1.00	1.00	1.00		1.00
Primary school	1.10 (0.97, 1.24)	1.06 (0.93, 1.20)	0.91 (0.76, 1.11)	0.90 (0.74, 1.09)	0.84 (0.79, 0.90)^c^	0.86 (0.80, 0.93)^c^	0.70 (0.63, 0.77)^c^	0.71 (0.64, 0.79)^c^
Secondary school	1.34 (1.19, 1.52)^c^	1.22 (1.08, 1.39)^b^	1.14 (0.95, 1.37)	1.03 (0.85, 1.24)	0.81 (0.75, 0.86)^c^	0.81 (0.75, 0.88)^c^	0.49 (0.44, 0.54)^c^	0.48 (0.43, 0.55)^c^
High school and above	1.48 (1.30, 1.69)^c^	1.32 (1.15, 1.52)^c^	1.26 (1.03, 1.54)^a^	1.13 (0.91, 1.39)	0.57 (0.51, 0.63)^c^	0.58 (0.51, 0.65)^c^	0.31 (0.26, 0.38)^c^	0.32 (0.26, 0.39)^c^
Household size								
0–3	1.00	1.00	1.00	1.00	1.00	1.00		1.00
4–6	1.02 (0.95, 1.08)	0.99 (0.93, 1.06)	0.97 (0.88, 1.06)	0.98 (0.89, 1.09)	0.98 (0.93, 1.03)	1.00 (0.94, 1.06)	0.85 (0.79, 0.93)^c^	0.88 (0.80, 0.96)^b^
7–9	1.07 (0.93, 1.23)	1.05 (0.91, 1.22)	1.11 (0.89, 1.37)	1.15 (0.92, 1.43)	1.15 (1.01, 1.31)^a^	1.11 (0.97, 1.26)	1.48 (1.24, 1.75)^c^	1.32 (1.11, 1.58)^b^
Annual family income (CNY)								
< 29,999	1.00	1.00	1.00	1.00	1.00	1.00		1.00
30,000–59,999	1.05 (0.97, 1.13)	0.96 (0.89, 1.03)	1.09 (0.98, 1.23)	0.98 (0.87, 1.10)	1.01 (0.95, 1.08)	1.03 (0.96, 1.10)	0.88 (0.80, 0.97)^a^	0.94 (0.85, 1.04)
≥ 60,000	1.09 (1.01, 1.17)^a^	0.96 (0.88, 1.04)	0.90 (0.81, 1.02)	0.78 (0.69, 0.88)^c^	0.81 (0.76, 0.86)^c^	0.86 (0.80, 0.92)^c^	0.63 (0.57, 0.70)^c^	0.72 (0.65, 0.81)^c^
Smoking								
No	1.00	1.00	1.00	1.00	1.00	1.00		1.00
Yes	0.75 (0.71, 0.80)^c^	0.70 (0.66, 0.75)^c^	0.68 (0.61, 0.75)^c^	0.62 (0.56, 0.69)^c^	0.86 (0.74, 1.00)	0.77 (0.66, 0.89)^b^	1.11 (0.90, 1.36)	0.84 (0.68, 1.04)
Drinking								
No	1.00	1.00	1.00	1.00	1.00	1.00		1.00
Yes	1.15 (1.08, 1.22)^c^	1.28 (1.19, 1.36)^c^	1.14 (1.04, 1.26)^b^	1.30 (1.17, 1.44)^c^	1.20 (1.01, 1.42)^a^	1.22 (1.03, 1.46)^a^	1.55 (1.23, 1.95)^c^	1.45 (1.14, 1.83)^b^
Fresh vegetables								
No	1.00	1.00	1.00	1.00	1.00	1.00		1.00
Yes	0.95 (0.81, 1.12)	0.94 (0.79, 1.11)	1.09 (0.84, 1.42)	1.06 (0.80, 1.39)	1.06 (0.92, 1.23)	0.93 (0.79, 1.08)	1.07 (0.86, 1.34)	1.03 (0.82, 1.31)
Fresh fruit								
No	1.00	1.00	1.00	1.00	1.00	1.00		1.00
Yes	1.02 (0.96, 1.08)	1.01 (0.94, 1.09)	1.01 (0.92, 1.11)	0.91 (0.81, 1.01)	1.12 (1.06, 1.18)^c^	1.04 (0.97, 1.12)	0.94 (0.86, 1.02)	0.93 (0.84, 1.03)
Meat, egg and milk								
No	1.00	1.00	1.00	1.00	1.00	1.00		1.00
Yes	0.99 (0.93, 1.06)	0.91 (0.84, 1.00)^a^	1.09 (0.98, 1.21)	0.94 (0.82, 1.07)	1.23 (1.16, 1.31)^c^	1.15 (1.06, 1.24)^c^	1.20 (1.10, 1.31)^c^	1.17 (1.04, 1.31)^b^
Soy products								
No	1.00	1.00	1.00	1.00	1.00	1.00		1.00
Yes	1.02 (0.96, 1.09)	1.01 (0.93, 1.09)	1.18 (1.06, 1.30)^b^	1.14 (1.00, 1.29)^a^	1.22 (1.15, 1.29)^c^	1.11 (1.03, 1.20)^b^	1.20 (1.10, 1.31)^c^	1.13 (1.02, 1.26)^a^
Pickled food								
No	1.00	1.00	1.00	1.00	1.00	1.00		1.00
Yes	1.27 (1.17, 1.38)^c^	1.30 (1.18, 1.42)^c^	1.65 (1.47, 1.86)^c^	1.62 (1.42, 1.84)^c^	1.25 (1.16, 1.35)^c^	1.13 (1.04, 1.22)^b^	1.77 (1.60, 1.96)^c^	1.46 (1.30, 1.63)^c^
Fried food								
No	1.00	1.00	1.00	1.00	1.00	1.00		1.00
Yes	1.07 (0.90, 1.27)	0.91 (0.76, 1.09)	1.27 (0.99, 1.62)	0.92 (0.71, 1.20)	1.22 (1.04, 1.44)^a^	1.07 (0.90, 1.27)	1.37 (1.09, 1.72)^b^	1.00 (0.78, 1.27)
Hot food								
No	1.00	1.00	1.00	1.00	1.00	1.00		1.00
Yes	1.20 (1.05, 1.37)^b^	1.12 (0.97, 1.29)	1.46 (1.21, 1.76)^c^	1.22 (1.00, 1.49)^a^	1.24 (1.11, 1.39)^c^	1.12 (0.99, 1.26)	1.55 (1.32, 1.81)^c^	1.16 (0.99, 1.37)
Mouldy food								
No	1.00	1.00	1.00	1.00	1.00	1.00		1.00
Yes	1.05 (0.61, 1.82)	0.97 (0.55, 1.70)	1.09 (0.47, 2.51)	0.88 (0.37, 2.07)	0.79 (0.46, 1.34)	0.66 (0.38, 1.13)	1.02 (0.49, 2.12)	0.74 (0.35, 1.57)

Regressions were adjusted for age, marital status, education, household size, annual family income, smoking, drinking, intake of fresh vegetables, fresh fruit, meat, egg and milk, soy products, pickled food, fried food, hot food, and mouldy food. Adopting the Chinese standard (overweight: 24 kg/m^2^ ≤ BMI < 28 kg/m^2^; Obesity: BMI ≥ 28 kg/m^2^).

*COR* crude odds ratio, *AOR* adjusted odds ratio, *CI* confidence interval, *CNY* Chinese Yuan.

^a^*P* < 0.05. ^b^*P* < 0.01. ^c^*P* < 0.001. ^d^Never married/divorced/separated/widowed.

### Factors for overweight/obesity among different age groups

Variables associated with overweight or obesity in the final models included education, household size, annual family income, smoking, drinking, and intake of fresh vegetables, fruit and pickled food among both younger participants (aged 40 to 59 years) and older ones (aged 60 to 69 years) (Table 5). Meanwhile, the odds of being overweight and obese were lower among women aged 40 to 59 years (overweight: AOR = 0.68, 95% CI 0.64, 0.72; obesity: AOR = 0.68, 95% CI 0.62, 0.75). Married participants aged 60 to 69 years were more likely to be overweight (AOR = 1.12, 95% CI 1.00, 1.25) than their counterparts. Participants aged 40 to 59 years consuming the products of meat, egg, and milk were more likely to be overweight and obese than their counterparts (overweight: AOR = 1.08, 95% CI 1.00, 1.17; obesity: AOR = 1.20, 95% CI 1.07, 1.35). Intake of soy products (AOR = 1.17, 95% CI 1.05, 1.30) and hot food (AOR = 1.19, 95% CI 1.01,1.41) were the risk factors for obesity among individuals aged 40 to 59 years. Besides, we also found some patterns interesting from the results mentioned above. Intake of fresh vegetables was associated with being obese among individuals aged 60 to 69 years, while it had a protective effect among those aged 40 to 59 years. Interestingly, the effect of fruit intake in both populations was reversed (all *P* < 0.05).

**Table 5:** Factors associated with the prevalence of overweight or obesity among participants aged 40–59 and 60–69 years.

Variable	40–59 years				60–69 years			
	Overweight		Obesity		Overweight		Obesity	
	COR (95%CI)	AOR (95%CI)	COR (95%CI)	AOR (95%CI)	COR (95%CI)	AOR (95%CI)	COR (95%CI)	AOR (95%CI)
Gender								
Male	1.00	1.00	1.00	1.00	1.00	1.00	1.00	1.00
Female	0.74 (0.71, 0.78)^c^	0.68 (0.64, 0.72)^c^	0.82 (0.76, 0.89)^c^	0.68 (0.62, 0.75)^c^	1.04 (0.98, 1.11)	0.99 (0.91, 1.07)	1.31 (1.19, 1.44)^c^	1.07 (0.95, 1.21)
Marital status								
Other^d^	1.00	1.00	1.00	1.00	1.00	1.00	1.00	1.00
Married	1.10 (0.95, 1.27)	1.14 (0.99, 1.32)	0.98 (0.79, 1.21)	1.12 (0.90, 1.39)	1.13 (1.01, 1.26)^a^	1.12 (1.00, 1.25)^a^	1.04 (0.88, 1.23)	1.10 (0.93, 1.31)
Education								
Illiterate or semi-illiterate	1.00	1.00	1.00	1.00	1.00	1.00	1.00	1.00
Primary school	0.80 (0.72, 0.88)^c^	0.79 (0.72, 0.87)^c^	0.58 (0.51, 0.66)^c^	0.62 (0.54, 0.71)^c^	0.90 (0.84, 0.97)^b^	0.91 (0.84, 0.99)^a^	0.69 (0.61, 0.77)^c^	0.75 (0.66, 0.84)^c^
Secondary school	0.85 (0.78, 0.94)^b^	0.78 (0.71, 0.86)^c^	0.49 (0.44, 0.56)^c^	0.48 (0.43, 0.55)^c^	0.96 (0.88, 1.05)	0.97 (0.88, 1.07)	0.67 (0.59, 0.77)^c^	0.78 (0.67, 0.90)^b^
High school and above	0.80 (0.71, 0.88)^c^	0.70 (0.62, 0.78)^c^	0.45 (0.38, 0.52)^c^	0.43 (0.36, 0.50)^c^	1.04 (0.92, 1.17)	1.03 (0.91, 1.17)	0.76 (0.63, 0.92)^b^	0.87 (0.71, 1.06)
Household size								
0–3	1.00	1.00	1.00	1.00	1.00	1.00	1.00	1.00
4–6	0.99 (0.94, 1.04)	1.01 (0.96, 1.07)	0.93 (0.86, 1.01)	0.96 (0.88, 1.04)	0.98 (0.92, 1.05)	0.99 (0.92, 1.06)	0.84 (0.76, 0.93)^b^	0.88 (0.79, 0.98)^a^
7–9	1.25 (1.10, 1.43)^b^	1.21 (1.06, 1.38)^b^	1.52 (1.28, 1.82)^c^	1.38 (1.15, 1.65)^b^	0.97 (0.84, 1.12)	0.96 (0.83, 1.11)	1.10 (0.90, 1.35)	1.12 (0.90,1.38)
Annual family income (CNY)								
< 29,999	1.00	1.00	1.00	1.00	1.00	1.00	1.00	1.00
30,000–59,999	0.99 (0.93, 1.06)	0.97 (0.91, 1.04)	0.90 (0.81, 0.99)^a^	0.90 (0.82, 1.00)^a^	1.02 (0.95, 1.10)	1.01 (0.93, 1.09)	0.98 (0.88, 1.10)	1.01 (0.90, 1.14)
≥ 60,000	0.87 (0.82, 0.93)^c^	0.87 (0.81, 0.93)^c^	0.68 (0.61, 0.75)^c^	0.72 (0.64, 0.79)^c^	0.97 (0.90, 1.04)	0.95 (0.87, 1.03)	0.76 (0.67, 0.85)^c^	0.79 (0.69, 0.90)^b^
Smoking								
No	1.00	1.00	1.00	1.00	1.00	1.00	1.00	1.00
Yes	1.02 (0.95, 1.09)	0.72 (0.66, 0.78)^c^	0.96 (0.86, 1.06)	0.66 (0.58, 0.75)^c^	0.78 (0.72, 0.83)^c^	0.70 (0.65, 0.76)^c^	0.64 (0.57, 0.72)^c^	0.63 (0.55, 0.72)^c^
Drinking								
No	1.00	1.00	1.00	1.00	1.00	1.00	1.00	1.00
Yes	1.37 (1.27, 1.47)^c^	1.30 (1.19, 1.42)^c^	1.41 (1.27, 1.57)^c^	1.46 (1.29, 1.66)^c^	1.09 (1.01, 1.18)^a^	1.26 (1.15, 1.37)^c^	0.91 (0.80, 1.02)	1.15 (1.00, 1.33)
Fresh vegetables								
No	1.00	1.00	1.00	1.00	1.00	1.00	1.00	1.00
Yes	0.89 (0.77, 1.03)	0.80 (0.69, 0.93)^b^	0.93 (0.75, 1.15)	0.84 (0.67, 1.05)	1.20 (1.01, 1.42)^a^	1.16 (0.97, 1.38)	1.35 (1.02, 1.78)^a^	1.44 (1.07, 1.92)^a^
Fresh fruit								
No	1.00	1.00	1.00	1.00	1.00	1.00	1.00	1.00
Yes	1.09 (1.03, 1.15)^b^	1.08 (1.01, 1.15)^a^	0.99 (0.91, 1.08)	0.95 (0.86, 1.05)	1.01 (0.95, 1.08)	0.97 (0.90, 1.05)	0.91 (0.83, 1.01)	0.88 (0.79, 0.99)^a^
Meat, egg and milk								
No	1.00	1.00	1.00	1.00	1.00	1.00	1.00	1.00
Yes	1.17 (1.10, 1.24)^c^	1.08 (1.00, 1.17)^a^	1.28 (1.17, 1.40)^c^	1.20 (1.07, 1.35)^b^	1.04 (0.98, 1.12)	0.99 (0.91, 1.08)	0.98 (0.89, 1.09)	0.92 (0.81, 1.05)
Soy products								
No	1.00	1.00	1.00	1.00	1.00	1.00	1.00	1.00
Yes	1.15 (1.09, 1.22)^c^	1.07 (0.99, 1.15)	1.27 (1.16, 1.38)^c^	1.17 (1.05, 1.30)^b^	1.08 (1.01, 1.16)^a^	1.06 (0.98, 1.15)	1.08 (0.97, 1.20)	1.08 (0.96, 1.23)
Pickled food								
No	1.00	1.00	1.00	1.00	1.00	1.00	1.00	1.00
Yes	1.36 (1.26, 1.46)^c^	1.26 (1.16, 1.36)^c^	1.87 (1.69, 2.07)^c^	1.56 (1.40, 1.74)^c^	1.15 (1.05, 1.25)^b^	1.12 (1.02, 1.23)^a^	1.54 (1.36, 1.74)^c^	1.48 (1.30, 1.69)^c^
Fried food								
No	1.00	1.00	1.00	1.00	1.00	1.00	1.00	1.00
Yes	1.23 (1.05, 1.44)^b^	1.00 (0.85, 1.18)	1.47 (1.19, 1.82)^c^	1.00 (0.80, 1.26)	1.06 (0.89, 1.27)	0.97 (0.80, 1.18)	1.14 (0.87, 1.49)	0.89 (0.67, 1.19)
Hot food								
No	1.00	1.00	1.00	1.00	1.00	1.00	1.00	1.00
Yes	1.26 (1.12, 1.41)^c^	1.11 (0.98, 1.25)	1.61 (1.38, 1.88)^c^	1.19 (1.01, 1.41)^a^	1.17 (1.03, 1.34)^a^	1.12 (0.97, 1.28)	1.38 (1.14, 1.67)^b^	1.16 (0.95, 1.42)
Mouldy food								
No	1.00	1.00	1.00	1.00	1.00	1.00	1.00	1.00
Yes	1.02 (0.62, 1.68)	0.85 (0.52, 1.41)	1.02 (0.49, 2.15)	0.73 (0.34, 1.54)	0.77 (0.42, 1.39)	0.72 (0.39, 1.33)	1.09 (0.47, 2.50)	0.97 (0.41, 2.27)

Regressions were adjusted for gender, marital status, education, household size, annual family income, smoking, drinking, intake of fresh vegetables, fresh fruit, meat, egg and milk, soy products, pickled food, fried food, hot food, and mouldy food. Adopting the Chinese standard (Overweight: 24 kg/m^2^ ≤ BMI < 28 kg/m^2^; Obesity: BMI ≥ 28 kg/m^2^).

*COR* crude odds ratio, *AOR* adjusted odds ratio, *CI* confidence interval, *CNY* Chinese Yuan.

^a^*P* < 0.05. ^b^*P* < 0.01. ^c^*P* < 0.001. ^d^Never married/divorced/separated/widowed.

Finally, we re-performed the final model using the WHO standard criteria for overweight/obesity among the whole population as a robustness check (Supplementary Table 2). The results showed that some variables produced changes in predicting overweight or obesity. However, there was no substantial change in the qualitative conclusions except for variables such as intake of fresh fruit, meat, egg and dairy, and mouldy food.

## Discussion

To our knowledge, this is the first study based on a large representative sample to investigate the prevalence of overweight and obesity and its influencing factors and examine whether the association varied across different subgroups among residents aged 40–69 years in high-risk areas of UGC in Jiangsu Province, southeastern China. In this study, we found that over half of the participants were overweight or obese by Chinese standards. The overweight or obesity rate was also close to 40% when using WHO standards. Being female, having a higher education level, having a higher annual family income, smoking, and having fresh fruit were independent protective factors for overweight or obesity. Being married, drinking, soy products, pickled and hot food intake were independent risk factors for overweight or obesity. Meanwhile, the odds of being overweight or obese tended to rise and then fall with increasing age, while the trend was reversed between household size and overweight or obesity. Subgroup analyses revealed differences in the role of age, education, and meat, egg and milk intake on overweight or obesity between men and women. Besides, there were also differences in the role of fresh vegetables and fruit intake on overweight or obesity between the younger (40–59 years) and older (60–69 years) groups.

Based on the Chinese standards, the prevalence of overweight and obesity in our study was 42.1% and 11.9%, respectively. Using WHO standards, the prevalence of overweight was 34.7%, and obesity was 4.7%. Data from several studies from different regions based on Chinese standards indicated that the prevalence of overweight among adults in the northwest (aged 35+ years), southern (aged 15+ years), northeast (aged 18–79 years) was 36.5%, 25.8% and 32.3%, and the prevalence of obesity was 26.5%, 7.9% and 14.6%, respectively^4,19,26^. The prevalence of overweight in this study was at high levels, and obesity was at moderate levels, which was further confirmed in studies that used WHO standards^27,28^. Besides, the overall prevalence of overweight and obesity (54% for Chinese standards and 39.4% for WHO standards) in our study was higher than what was reported in and outside of China^19,26–29^. This finding indicated that the overall prevalence of overweight and obesity among high-risk participants at risk of UGC is significant and that disease control authorities need to strengthen weight interventions for target populations in addition to conventional cancer prevention measures. Meanwhile, we found that the prevalence of overweight was higher in men than women, consistent with other studies^4,19,26^. Women had higher rates of obesity than men, consistent with Kaboré et al.^29^ and Oguoma et al.^30^, but inconsistent with other studies^4,19,26,28^. This difference may be attributed to the difference in respondents' age, lifestyle, and economic level in various regions^19,26^.

We found that sociodemographic variables, including gender, age, marital status, education, household size and annual family income, were associated with being overweight or obese among participants. The odds of being overweight or obese were about 0.8 times among females than males. This is expected because of the apparent differences in living and eating habits between men and women^19^. 50–69 years of females seemed to be the risk group for being overweight compared to 40–49 years, consistent with other studies^28,29^. With increasing age among females, changes in estrogen, decreased physical activity, and specific cultural beliefs may have contributed to this result^29,31,32^. Nevertheless, when men reached the age of 60–69 years, age appeared to be a factor in weight loss, which was inconsistent with other studies^28,29,33^. The possible explanation could be that older men in rural areas are often accompanied by multiple chronic diseases and lower socioeconomic status, which leads to weight loss. On the other hand, rural elderly inhabitants are predominantly manual workers, which makes them less prone to over-nutrition. Married participants had higher odds of being overweight than those with other marital statuses, especially among men and participants aged 60 to 69 years. Although the specific mechanisms by which marital status contributes to weight gain in men are unclear, weight gain appears to be a common phenomenon as men enter marriage, which may be related to the stability of life after marriage and the elimination of focusing on personal image^4,26^.

We found that education was negatively associated with overweight/obesity among participants. When stratified by gender, the relationship between education and overweight or obesity was reversed. Specifically, increasing educational attainment increased the risk of being overweight in men but decreased the risk of being overweight or obese in women. Tekalegn et al.^34^ in Ethiopia found that risks among men with higher education levels were 1.6 ~ 3.6 times higher than men with no education. Kaboré et al.^29^, in a cross-section study conducted in Burkina Faso, also noted a potential increased risk among men. Meanwhile, some studies have confirmed the negative association between education and overweight/obesity among females^35,36^. However, in some studies from other countries, we can see a positive association between them^37,38^. Education is one of the indicators of socioeconomic status. A good education means having a better socioeconomic status and thus showing advantages in health literacy and access to health care, especially in rural areas^22,39^. This is a promising finding, as good resource advantages in women have translated into the preventive behaviour of overweight and obesity, unlike the males. It reminds us that men with higher education levels should be the focus population for weight management in the future intervention process. This finding was further confirmed by another indicator of socioeconomic status (annual family income) in this study. Specifically, participants with an annual family income of over 60,000 CNY were less likely to be overweight/obese among all subgroups.

We found a 'U' shaped risk of obesity as the number of family members increased. Specifically, compared to participants with 0–3 family members, the odds of obesity decreased when the household size was 4–6. However, the risk of obesity increased when the number of family members reached 7–9. The structure of a family's diet will change with the number of family members, with larger families meaning that food may be more diversified. Besides, social support among family members may also correct poor lifestyles^22^. However, the diet may be more sumptuous if participants have 7–9 family members. Due to the Chinese tradition of saving, any excess food will be consumed by adults, which increases the risk of over-nutrition. Some researchers have also reported that household size is negatively associated with leisure-time physical activity, increasing the risk of weight gain^40^.

We also found that living and dietary habits, including smoking, drinking, fresh fruit intake, soy products intake, and pickled and hot food intake, were associated with being overweight or obese among participants. Smokers were less likely to be overweight/obese, consistent with previous studies^4,26,28,29^. This can be explained by the loss of appetite associated with smoking and the weight loss caused by smoking-related diseases. The positive association between alcohol consumption and overweight/obesity is also reported in some literature mentioned above^4,26^. Drinking behaviour in China is usually accompanied by a high intake of high-salt and high-fat foods^4,26^. Furthermore, influenced by traditional culture, it is commonly believed that the more one drinks, the deeper the bond between drinkers, which leads to the fact that the more one drinks, the more likely one is to be obese^26^. In our study, fresh fruit intake was a protective factor against obesity, while fresh vegetable intake had no significant impact on overweight or obesity. When stratified by age, both were meaningful for overweight or obese but performed oppositely. The intake of vegetables and fruit, as one of the diets recommended by the Dietary Guidelines, is often seen as one of the measures to improve weight. Buijsse and colleagues observed a weak association between fruit and vegetable intake and weight loss^41^. Another study in the USA also reported that for most older people in urban areas, there was a significant negative association between obesity and the consumption of fruit and green vegetables^42^. However, a study has also found that vegetable-rich food patterns were associated with obesity/centripetal obesity in Chinese adults, which they attributed to the high energy content of a large amount of oil used to cook vegetables^43^. The soy protein and isoflavones in soy foods benefit many chronic diseases, including obesity, which has been reported in several studies^44,45^, inconsistent with our findings. This phenomenon may be related to how soy products are cooked. For example, soy products such as tofu, fish tofu and almond tofu are high in sugars, oils and fats as well as sodium, which undoubtedly increases the risk of calorie overload.

We found that participants eating pickled food were more likely to be overweight and obese. Traditionally in China, pickled food is prepared by soaking foods in high salt levels over a long time. Along with the intake of pickled foods, large amounts of salt are consumed, increasing the risk of obesity^46,47^. Meanwhile, a Chinese study reported that pickled products might increase caloric intake as an appetite booster^48^. Being overweight and obese was more likely associated with a regular intake of hot food. There may be several reasons: Firstly, hot food is too scalding to chew adequately in the mouth, leading to eating too quickly. However, eating too fast is less likely to produce a feeling of satiety and thus increase food consumption per unit of time. Secondly, hot food causes the blood vessels in the intestine to dilate and the digestive glands to become more active, significantly increasing digestion and absorption^49,50^. We also found that the effect of meat, egg and dairy on overweight/obesity was different between men and women. A possible explanation is that meat, egg and dairy products were not finely differentiated in this study to identify the frequency of intake of specific foods among them. If participants consume meat in excess, it can increase the risk of being overweight/obese^51,52^. However, the intake of egg and dairy products may moderate poor body weight^53,54^.

This study provides a valuable perspective on preventing UGC in high-risk populations. The UGC risk may be reduced if the weight assessment of participants and obesity prevention strategies are emphasized during UGC screening at screening centres. The current screening strategy for UGC should undoubtedly be maintained and strengthened because of its demonstrated effectiveness in cancer prevention and control^55,56^. However, cancer screening focuses more on clinical outcomes and less on obesity-related health education and behavioural interventions for participants. Therefore, staff first need to be more aware of the importance of obesity in reducing cancer risk. Particular attention should be paid to overweight, obese and even severely obese participants. A course on obesity management should be included in regular training to assess participants’ obesity better and to provide timely weight interventions during screening or refer subjects to family doctors for further help.

Participants may have warning signals of obesity regarding basic characteristics, diet or lifestyle. Focusing on different warning signals and their patterns can help staff to more accurately identify participants at high risk of obesity for early intervention. Care should be taken to identify and mark a screening participant if someone is male, married, consumes alcohol or prefers pickled and hot food. In addition to interventions during the screening process, targeted follow-up services should be considered. At the screening return visit, obese participants should be reminded about changing their poor lifestyle and asked about their current weight change. If there is a need for greater concern, they can be advised to a screening centre or hospital for health guidance and health care services. The effectiveness of interventions can be improved by drawing on several well-established behavioural theory models, including the theory of planned behaviour and the transtheoretical model^57,58^.

This is the first large sample study of overweight and obesity in the population aged 40–69 years in areas at high risk of UGC. The data involved are reliable because they were collected by trained staff after following a standard technical protocol. However, our study also has some limitations because of its secondary analysis of previous data. Firstly, we did not obtain a variable for physical activity, an indicator strongly associated with obesity. Secondly, we only utilised BMI to assess obesity and could not address central obesity, as it was unavailable in the Rural Early Diagnosis and Treatment of UGC dataset. Again, except for height and weight, all survey items were obtained through self-reporting by participants, which may be subject to recall and reporting bias. Finally, this study is a typical cross-sectional study. Therefore, it was impossible to determine the causal relationship between overweight/obesity and the factors studied.

In conclusion, our study reveals a high burden of overweight and obesity among adults aged 40–69 years from the high-risk regions of UGC in Jiangsu Province, southeastern China. Risk factors affecting overweight or obesity include male, younger age, married status, lower education level, larger household size, lower annual family income, non-smoking, drinking, lower fresh fruit intake, and intake of soy products, pickled, and hot food. However, these influencing factors may vary by gender and age group. Our study highlights the importance of individualised interventions to improve the weight of high-risk groups for UGC, except for conventional cancer prevention measures.

## Supplementary Information

Supplementary Tables.

## Data Availability

The datasets generated during and/or analysed during the current study are available from the corresponding author on reasonable request.
