# Supplementary material for: The prevalence and determinant of overweight and obesity among residents aged 40–69 years in high-risk regions for upper gastrointestinal cancer in southeast China
Source: Sci Rep. 2023 May 20;13:8172. doi: 10.1038/s41598-023-35477-x (PMC10199934; doi:10.1038/s41598-023-35477-x)
Supplement: Supplementary file 1 — Supplementary Tables. [file 41598_2023_35477_MOESM1_ESM.docx]

**The prevalence and determinant of overweight and obesity among residents aged 40-69 years in high-risk regions for Upper gastrointestinal cancer in southeast China**

Xiang Feng^1^, Jinhua Zhu^1,3,*,+^, Zhaolai Hua^1^, Qiuping Shi^1^, Jinyi Zhou^2,*,+^ & Pengfei Luo^2^

^1^Institute of Tumour Prevention and Control, Yangzhong People’s Hospital, Yangzhong, 212200, China

^2^Department of Non-communicable Disease Prevention and Control, Jiangsu Provincial Centre for Disease Control and Prevention, Nanjing, 210009, China

^3^Department of Gastroenterology, Zhongda Hospital, Southeast University, Nanjing, 210000 China

^*^zhujinhuavip@sina.com; [zhoujinyi74@sina.com](mailto:zhoujinyi74@sina.com)

^+^These authors contributed equally to this work

| **Name:** |  |  |  |  |  |
| --- | --- | --- | --- | --- | --- |
| **ID:** |  |  |  |  |  |
| **Sex (1=Male, 2=Female):** |  |  |  |  |  |
| **Date of birth:** |  |  |  |  |  |
| **ID card:** |  |  |  |  |  |
| **Home Address:** |  | **Code:** |  | **Contact number:** |  |
| **Investigator:** |  |  |  |  |  |
| **Survey date:** |  |  |  |  |  |
| 1. **General information** |  |  |  |  | q1 |
| 1.1 Marital status (0=Single, 1=Currently married, 2=Divorced, 3=Widowed) |  |  |  |  | q1-1 |
| 1.2 Education (0=No institutional education, 1=Primary school, 2=Junior high school, 3=Senior high school, 4= College and higher) |  |  |  |  | q1-2 |
| 1.3 How many people are there in your family? |  |  |  |  | q1-3 |
| 1.4 What is the average annual income of your family (CNY)? |  |  |  |  | q1-4 |
| 1. **Physical examination results** |  |  |  |  | q2 |
| 2.1 Height (cm) |  |  |  |  | q2-1 |
| 2.2 Weight (kg) |  |  |  |  | q2-2 |
| 2.3 Pulse (times/minute) |  |  |  |  | q2-3 |
| 2.4 Blood pressure (mmHg) |  |  |  |  | q2-4 |
| 1. **Smoking (0=No, 1=Yes)** |  |  |  |  | q3 |
| **Types** | **Yes or No** | **Do you smoke every day?**  **(0=Yes, 1=No)** | **How much do you smoke daily or monthly?** | **How many years have you smoked?** |  |
| 3.1 Cigarette (daily) | q3-1a | q3-1b | q3-1c | q3-1d | q3-1 |
| 3.2 Tobacco leaf (liang/per month) | q3-2a | q3-2b | q3-2c | q3-2d | q3-2 |
| **4. Drinking (0=No, 1=Yes)** |  |  |  |  | q4 |
| **Types** | **Yes or No** | **Do you drink every day?**  **(0=Yes, 1=No)** | **How much alcohol do you drink every day?** | **How many years have you drunk?** |  |
| 4.1 Regardless of the type of alcohol (liang/daily) | q4-1a | q4-1b | q4-1c | q4-1d | q4-1 |
| **5. Eating habits (In the past year, have you eaten the following foods?)** |  |  |  |  | q5 |
| **Types** | **Never** | **Seldom (< twice/week)** | **Often (≥ twice/week)** | |  |
| 5.1 Fresh vegetables | q5-1a | q5-1b | q5-1c | | q5-1 |
| 5.2 Fresh fruit | q5-2a | q5-2b | q5-2c | | q5-2 |
| 5.3 Meat, egg and dairy products | q5-3a | q5-3b | q5-3c | | q5-3 |
| 5.4 Soy products | q5-4a | q5-4b | q5-4c | | q5-4 |
| 5.5 Pickled food | q5-5a | q5-5b | q5-5c | | q5-5 |
| 5.6 Fried food | q5-6a | q5-6b | q5-6c | | q5-6 |
| 5.7 Hot food | q5-7a | q5-7b | q5-7c | | q5-7 |
| 5.8 Mouldy food | q5-8a | q5-8b | q5-8c | | q5-8 |
| **6. History of digestive system disorders (0=No, 1=Yes)** |  |  |  | | q6 |
| **Name of disease** |  | **Yes or No** | **Age at diagnosis (year)** | |  |
| 6.1 Gastroenteritis |  |  |  | | q6-1 |
| 6.2 Gastric and duodenal ulcers |  |  |  | | q6-2 |
| 6.3 Esophagitis |  |  |  | | q6-3 |
| 6.4 Hepatitis |  |  |  | | q6-4 |
| 6.5 Others, please specify |  |  |  | | q6-5 |

**Table 1.** Basic information questionnaire. 1 liang=50g. *CNY* China yuan.

| **Variable** | **Overweight** | | **Obesity** | |
| --- | --- | --- | --- | --- |
|  | **AOR (95%CI)** | ***P*-value** | **AOR (95%CI)** | ***P*-value** |
| Gender |  |  |  |  |
| Male | 1.00 |  | 1.00 |  |
| Female | 0.80(0.76,0.84) | < 0.001 | 0.95(0.85,1.07) | 0.387 |
| Age group (years) |  |  |  |  |
| 40-49 | 1.00 |  | 1.00 |  |
| 50-59 | 1.05(0.99,1.11) | 0.092 | 0.99(0.87,1.11) | 0.812 |
| 60-69 | 0.86(0.81,0.91) | < 0.001 | 0.71(0.62,0.81) | < 0.001 |
| Marital Status |  |  |  |  |
| Other^a^ | 1.00 |  | 1.00 |  |
| Married | 1.11(1.02,1.22) | 0.019 | 1.12(0.92,1.36) | 0.277 |
| Education |  |  |  |  |
| Illiterate or semi-illiterate | 1.00 |  | 1.00 |  |
| Primary school | 0.78(0.73,0.83) | < 0.001 | 0.62(0.55,0.71) | < 0.001 |
| Secondary school | 0.74(0.69,0.79) | < 0.001 | 0.49(0.43,0.56) | < 0.001 |
| High school and above | 0.73(0.68,0.79) | < 0.001 | 0.48(0.40,0.57) | < 0.001 |
| Household size |  |  |  |  |
| 0-3 | 1.00 |  | 1.00 |  |
| 4-6 | 1.00(0.96,1.04) | 0.987 | 0.95(0.86,1.04) | 0.272 |
| 7-9 | 1.19(1.09,1.31) | < 0.001 | 1.28(1.05,1.56) | 0.014 |
| Family yearly income (CNY) |  |  |  |  |
| < 29,999 | 1.00 |  | 1.00 |  |
| 30,000-59,999 | 0.97(0.93,1.02) | 0.282 | 0.94(0.84,1.04) | 0.224 |
| ≥ 60,000 | 0.83(0.79,0.88) | < 0.001 | 0.66(0.59,0.75) | < 0.001 |
| Smoking |  |  |  |  |
| No | 1.00 |  | 1.00 |  |
| Yes | 0.76(0.71,0.80) | < 0.001 | 0.67(0.58,0.77) | < 0.001 |
| Drinking |  |  |  |  |
| No | 1.00 |  | 1.00 |  |
| Yes | 1.26(1.18,1.34) | < 0.001 | 1.39(1.21,1.60) | < 0.001 |
| Fresh vegetables |  |  |  |  |
| No | 1.00 |  | 1.00 |  |
| Yes | 0.96(0.85,1.07) | 0.442 | 1.09(0.84,1.41) | 0.527 |
| Fresh fruit |  |  |  |  |
| No | 1.00 |  | 1.00 |  |
| Yes | 0.97(0.92,1.02) | 0.247 | 0.93(0.83,1.03) | 0.174 |
| Meat, egg and milk |  |  |  |  |
| No | 1.00 |  | 1.00 |  |
| Yes | 1.06(1.00,1.12) | 0.049 | 1.08(0.95,1.23) | 0.250 |
| Soy products |  |  |  |  |
| No | 1.00 |  | 1.00 |  |
| Yes | 1.09(1.03,1.15) | 0.003 | 1.02(0.90,1.14) | 0.776 |
| Pickled food |  |  |  |  |
| No | 1.00 |  | 1.00 |  |
| Yes | 1.26(1.19,1.33) | < 0.001 | 1.48(1.31,1.67) | < 0.001 |
| Fried food |  |  |  |  |
| No | 1.00 |  | 1.00 |  |
| Yes | 0.96(0.85,1.08) | 0.506 | 1.00(0.78,1.29) | 0.997 |
| Hot food |  |  |  |  |
| No | 1.00 |  | 1.00 |  |
| Yes | 1.16(1.06,1.26) | 0.001 | 1.13(0.94,1.36) | 0.191 |
| Mouldy food |  |  |  |  |
| No | 1.00 |  | 1.00 |  |
| Yes | 0.67(0.45,0.99) | 0.045 | 0.74(0.32,1.72) | 0.483 |

**Table 2.** Factors associated with the prevalence of overweight or obesity among participants. Regressions were adjusted for gender, age, marital status, education, household size, annual family income, smoking, drinking, intake of fresh vegetables, fresh fruit, meat, egg and milk, soy product, pickled food, fried food, hot food, and mouldy food. Adopting the WHO standard (Overweight: 25 kg/m^2^ ≤ BMI < 30 kg/m^2^; Obesity: BMI ≥30 kg/m^2^). *COR* crude odds ratio. *AOR* adjusted odds ratio. *CI* confidence interval. *CNY* Chinese Yuan. ^a^ Never married/divorced/separated/widowed.
